# COVID-19 Challenges in Autoimmune Hepatitis Management: A Successful Outcome With Intravenous Immune Globulin (IVIg)

**DOI:** 10.1155/crgm/4446896

**Published:** 2025-04-07

**Authors:** Amirali Mashhadiagha, Parnian Khalili, Maryam Ataollahi, Fereshteh Karbasian, Ali Arman, Bita Geramizadeh

**Affiliations:** ^1^Shiraz Transplant Research Center, Shiraz University of Medical Sciences, Shiraz, Iran; ^2^Student Research Committee, Shiraz University of Medical Sciences, Shiraz, Iran; ^3^Department of Pediatric Gastroenterology, Shiraz University of Medical Sciences, Shiraz, Iran; ^4^Ali-Asghar Children's Hospital, Iran University of Medical Sciences, Tehran, Iran

**Keywords:** autoimmune, case report, COVID-19, hepatitis, immunoglobulins, intravenous, pediatrics

## Abstract

**Background:** Several autoimmune diseases, such as autoimmune hepatitis (AIH), can arise or become decompensated following COVID-19 infection or vaccination; however, there is a lack of data regarding the management of concurrent COVID-19 infection and autoimmune diseases.

**Case Summary:** In this paper, we present a case of a 9-year-old boy with yellowish discoloration of the skin and sclera, abnormal liver function test, followed by positive qRT-PCR for SARS-CoV-2 and progressive bicytopenia. After a lack of response to corticosteroids, intravenous immune globulin (IVIg) was administrated and a decline in liver enzymes, total bilirubin, and direct bilirubin was observed.

**Result and Discussion:** This case illustrates how IVIg significantly improved the AIH symptoms in the patient with positive qRT-PCR for the SARS-CoV-2 test. We hope our report encourages further research on therapeutic approaches for AIH concomitant with COVID-19.

## 1. Introduction

Autoimmune hepatitis (AIH) is a chronic, inflammatory liver disease that affects children and adults. AIH is classified into two main types: Type 1 and Type 2, based on specific autoantibodies. Although the cause of AIH is unknown, viruses and drugs may act as triggers in genetically predisposed individuals. AIH presents in a variety of ways. Fatigue, nausea, vomiting, jaundice, dark urine, pale stool, and fever are the common symptoms in the first group of patients. The second group presents with progressive fatigue, intermittent jaundice, and weight loss with a more gradual progression. In another form of AIH, the asymptomatic individual is diagnosed with high transaminase levels through routine laboratory tests. Furthermore, immunosuppression with corticosteroids is the conventional treatment for AIH [1].

The hepatitis A virus, hepatitis B virus, Epstein–Barr virus, and herpes simplex virus have all been linked to AIH [2]. Additionally, it has recently been reported that COVID-19 infection or vaccination can disrupt immunological tolerance, resulting in the onset or flare-up of immune-mediated diseases such as AIH [3, 4].

It is yet unclear how COVID-19 infection affects patients with AIH. Hence, definitive therapeutic approaches for these patients are not available. Multiple studies have found that AIH patients on immunosuppressive medication do not have an increased risk of severe COVID-19 infection. According to the American Association for the Study of Liver Diseases (AASLD) and the European Association for the Study of Liver (EASL), reduced immune modulation is not recommended without SARS-CoV-2 infection. However, in COVID-19 patients with severe illness, fever, lymphopenia, and bacterial, or fungal superinfection, lowering immunosuppressive treatment may be considered [5, 6].

Due to the advent of new SARS-CoV-2 mutations, and lack of data regarding management of the concurrent COVID-19 infection and autoimmune diseases, the COVID-19 pandemic remains a threat, and further studies are needed. Therefore, we provide a case of acute AIH in a child and the therapeutic challenges we faced following positive qRT-PCR for SARS-CoV-2 and progressive bicytopenia.

## 2. Case Presentation

A 9-year-old boy with an unremarkable past medical history, and no prior exposure to hepatotoxic medications, presented with yellowish discoloration of the skin and sclera, dark urine, clay-colored stool, nausea, vomiting, anorexia, fever, and abdominal distension. According to his mother, 3 weeks before this event, the whole family suffered from flank pain and dysuria, but they got better after a few days without taking any medication. There was no history of cough, headache, anosmia, sore throat, or dyspnea.

After developing icteric skin and sclera, he did a blood test that showed elevated values of alanine aminotransferase (ALT), aspartate aminotransferase (AST), and alkaline phosphatase (ALP). His jaundice got worse following an outpatient visit by a pediatrician. Thus, on the same day, 2 days after starting his signs and symptoms, he was admitted to Besat Hospital, Sanandaj, Iran, with the impression of acute hepatitis. qRT-PCR did not detect COVID-19, and laboratory tests for hepatitis A, B, C, and human immunodeficiency virus (HIV) were negative. Supportive therapy including ursodeoxycholic (UDCA) acid 525 mg/day tablet, 15 cc lactulose three times daily, and 15 mg famotidine twice daily and also 55 mcg vitamin K per day were started; however, the yellowish discoloration persisted, and liver enzymes remained high. In the meantime, to rule out other probable causes of acute hepatitis, such as AIH and Wilson's disease, additional tests were performed. Immunoglobulins were normal with an IgG of 641 mg/dL. Antismooth muscle antibody (Ab) and anti–liver–kidney microsomal Ab were negative. His serum ceruloplasmin level was 62 mg/dL, and his urinary copper level was 565 μg/day. After 7 days, without any change in his hospital drugs, the patient was discharged with no definite diagnosis; however, he was advised to continue his medication, including lactulose, famotidine, UDCA, and vitamin K until his follow-up visit scheduled for 1 week later. In addition, the family was told that if any serious symptoms appeared, they should go to the hospital right away.

**Figure 1:**
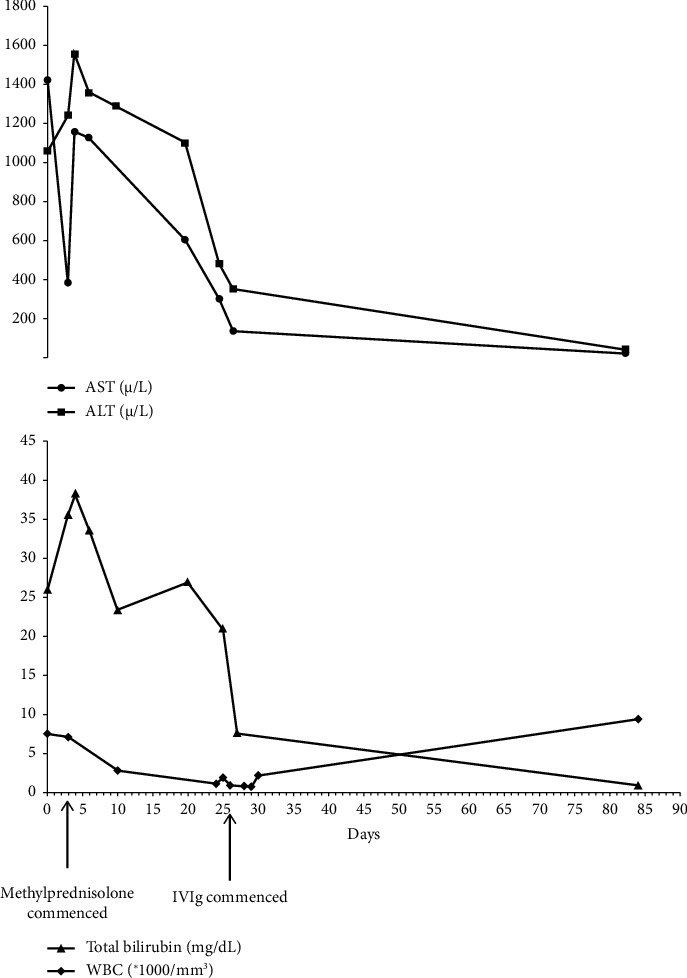
Fluctuations in total bilirubin, WBC, ALT, and AST according to time after presentation. Important therapies used for treatment are also shown.

The next day, he had one episode of nonbilious vomiting, so his parents brought him to the emergency department. He had a high fever (38.2°C) but no dyspnea. This time the qRT-PCR for SARS-CoV-2 became positive. There was a weak suspicion toward AIH; hence, the patient was administered methylprednisolone 40 mg/day, azathioprine 50 mg/day, and famotidine 30 mg/day who weighted 28 kg. Afterward, liver function tests and bilirubin levels were relatively improved. However, there was still no improvement in his skin color. Azathioprine 50 mg/day was adjusted to 100 mg/day on the third day of hospital admission. Furthermore, methylprednisolone 40 mg/day continued during the hospital course. After 5 days, he was discharged with prednisolone 25 mg/day and a recommendation for regular follow-up. The subsequent laboratory tests revealed deteriorating liver enzymes, total bilirubin, white blood cell (WBC), and red blood corpuscle (RBC) levels.  Figure 1 summarizes total bilirubin and WBC, ALT, and AST fluctuations over time.

After 13 days of his last hospitalization, he was transferred to Namazi Hospital, Shiraz, Iran, due to unresponsiveness to corticosteroids and progressive bicytopenia. In the emergency room, he presented with jaundice and reported a history of malaise along with severe fatigue upon exertion. His vital signs were blood pressure 110/80 mmHg, heart rate 110/min, respiratory rate 18/min, temperature 37°C, and Glasgow Coma Scale (GCS) 15/15. Laboratory investigation showed WBC = 1.1 × 1000/mm^3^, RBC = 4.1 Mill/mm^3^, platelet = 308 × 1000/mm^3^, AST = 300 µ/L, ALT = 480 µ/L, ALP = 572 µ/L, total bilirubin = 21 mg/dL, direct bilirubin = 15.7 mg/dL, PT = 14 s, and INR = 1.04. At physical examination, he had tender hepatomegaly (3 cm below the costal margin in mid-clavicular line), splenomegaly, icteric skin, and sclera. Then, he was transferred to the Pediatric Gastroenterology Ward, and qRT-PCR for COVID-19 turned out negative. Due to the increased 24-h urinary copper level in his previous laboratory tests which were taken 10 days after starting his symptoms in Sanandaj, an ophthalmologic consult was requested for him. However, the slit-lamp examination did not detect the Kayser–Fleischer ring, and Wilson's disease was ruled out. Additionally, we performed PCR testing for CMV and EBV, both of which returned negative results.

On the second day of Namazi Hospital admission, he presented with a fever (38.8°C), night sweats, and maculopapular rash throughout his body. Given the history of positive qRT-PCR for SARS-CoV-2, and with the impression of multisystem inflammatory syndrome in children (MIS-C), intravenous immunoglobulin (IVIg) 60 g/day was administered for 2 days, which led to recovery. Not only his fever and rash were resolved, but there was also a decline in liver enzymes, total bilirubin, and direct bilirubin. Simultaneously, methylprednisolone 60 mg/day was prescribed for 3 days.  Table 1 shows the laboratory values of the liver enzyme as well as total and direct bilirubin, albumin, and total protein after 2 days of IVIg 60 g/day administration in comparison with before administrations.

**Figure 2:**
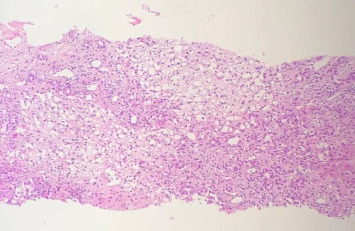
Liver histopathological findings. Submassive liver necrosis and moderate portal fibrosis with mild lymphocytic proliferation, stained with hematoxylin-eosin, magnification 100x.

Afterward, he was transferred to the Pediatric Immunology Ward for additional work-ups because of severely decreased RBC and WBC levels. Vancomycin 300 mg twice daily was initiated for probable bacterial infections. The peripheral blood smear revealed severe leukopenia, adequate platelet counts with some aggregation, and mild anisopoikilocytosis. Bone marrow aspiration showed moderate to severe hypocellular marrow, which was in favor of post–viral bone marrow suppression. Sections from the liver, stained with hematoxylin-eosin under a magnification of 100x, showed significant lymphoplasma cells infiltration both in the parenchyma and portal tracts with several foci of interface activity. Some hepatitis rosettes and foci of spotty and confluent necrosis are also present. Moreover, submassive liver necrosis and moderate portal fibrosis with mild lymphocytic proliferation were discovered during a liver biopsy in favor of AIH (Figure 2). Based on the simplified AIH scoring system, the following parameters were evaluated: ANA or SMA/F-actin were negative (zero points), LKM1 Ab was < 1 : 40 (zero points), SLA was negative (zero points), and IgG levels were within the normal range (zero points). Histological findings were compatible with AIH, contributing one point, and the absence of viral markers contributed two points. The total score was three, indicating a classification of possible AIH.

He got better in the following days, so he was discharged from the hospital with prednisolone 50 mg/day which was tapered by 10 mg each week over 6 weeks. After 6 weeks of treatment, the patient's liver enzymes and WBC level had returned to normal, and his symptoms had improved significantly.

## 3. Discussion

This report described a 9-year-old case without any significant past medical history that developed jaundice, abdominal distention, and elevated liver enzymes 3 weeks following a possible virus infection which affected the whole family. Twelve days following the onset of symptoms, qRT-PCR for the SARS-CoV-2 test became positive. The notable feature of our work is the simultaneousness of AIH with COVID-19 infection which dramatically responds to IVIg treatment. To our knowledge, this is the first report that demonstrates the efficacy of IVIg therapy in concordance with AIH and COVID-19.

AIH is a chronic liver disease with high morbidity and mortality if the diagnosis is delayed and immunosuppressive treatment is not started promptly [1, 2]. In our case, the patient dramatically responded to IVIg treatment. IVIg is a mixture of normal polyclonal IgG donated by thousands of healthy volunteers. It is frequently utilized in the treatment of autoimmune and inflammatory diseases. IVIg is indicated for treatment in some autoimmune diseases such as idiopathic thrombocytopenic purpura, Kawasaki disease, systemic lupus erythematosus (SLE), and chronic inflammatory demyelinating polyneuropathy (CIPD). For example, it has been shown that IVIg can reduce the formation of aneurysms in Kawasaki disease. In SLE, where corticosteroids are the first line of treatment, IVIg is used in major organ involvement such as pancytopenia, pneumonitis, pleural effusion, pericarditis, myocarditis, and nephritis. Several mechanisms of action have been proposed for explaining IVIg's immunomodulatory effects depending on the disease's nature [7].

On reviewing the literature, we identified a small number of reports on using IVIg in hepatitis treatment. Hsing et al. reported a kidney transplant patient who developed fulminant hepatitis due to the varicella-zoster virus. She was administrated with intravenous acyclovir and IVIg and improved without any complication [8]. Also, in a randomized clinical experiment involving 42 patients with chronic hepatitis C, a higher percentage of patients who received the combination of IVIg and interferon-α in comparison with patients who received IFN-α alone, exhibited virological and histological responses, suggesting an immunomodulating role for IVIg [9]. Moreover, Gupta et al. described a successful treatment with corticosteroids and IVIg in a 60-year-old woman, who developed syncytial giant cell hepatitis, a known case of chronic lymphocytic leukemia [10]. After comprehensive searching the literature about the administration of IVIg in AIH, we identified only a case that IVIg therapy was employed as a required alternative in a patient with immune-mediated chronic active hepatitis confirmed by histology in which long-term steroid treatment led to aseptic necrosis of the femur head. IVIg administration resulted in normalization of liver enzymes and clearance of circulating immune complexes. Also, histological examination of the liver showed a loss of periportal mononuclear cell infiltrates, with immunohistochemistry indicating the disappearance of intracellular IgG deposits [11].

In this case, our aim in using IVIG was to achieve two objectives simultaneously: to control both COVID-19 and AIH. We hypothesized that the reason AIH was not responding to multiple doses of corticosteroids was due to COVID-19 being involved in the disease process as studies have indicated that IVIg is effective in cases of severe COVID-19 in children [12, 13]. On reviewing the literature, we found a limited number of reports on AIH triggered by COVID-19. COVID-19 infection or vaccination has recently been linked to the onset or flare-up of AIH [3, 4]. However, in our case, the history of COVID-19 vaccination was negative. Mostafavi et al. [14] described a 13-year-old girl who developed simultaneous, new-onset AIH, celiac disease, and Hashimoto's thyroiditis following a COVID-19 infection. Memar et al. [15] reported an 11-year-old boy with positive qRT-PCR for SARS-CoV-2 that presented with fever, icterus, and abdominal pain. Zhou et al. [16] also described a case involving a 39-year-old woman who developed yellow skin and urine approximately 3 weeks after recovery from SARS-CoV-2 infection. After administration of methylprednisolone, she achieved recovery. In another study by Boettler et al. [17], the authors reported a 52-year-old male with episodes of AIH each after COVID-19 BNT162b2 mRNA vaccination. The liver biopsy revealed an immune infiltration quantitatively dominated by activated cytotoxic CD8 T-cells with panlobular distribution, elucidating distinct pathomechanism of COVID-19 vaccination-induced AIH. The patient received oral budesonide; however, his symptoms relapsed. After systemic steroid administration, he achieved remission. Another report by Ueno et al. [18] described a 54-year-old woman who developed AIH followed by receiving two doses of the Pfizer–BioNTech COVID-19 mRNA vaccine and an additional dose of the Moderna COVID-19 mRNA vaccine. The patient did not respond to corticosteroids. Consequently, azathioprine was added and liver biochemistry tests gradually improved. Their findings highlighted the efficacy of azathioprine for steroid-refractory AIH induced by COVID-19 vaccination. In our case, the liver biochemistry tests could not be improved by systemic steroids, and after IVIg administration, the liver biochemistry tests significantly improved. These findings suggest the consideration of alternative drugs when steroids cannot improve AIH caused by COVID-19 or COVID-19 vaccination. Also, according to the findings, considering COVID-19 infection at the onset of any acute hepatitis is recommended [3, 4, 15].

There are different theories about the underlying mechanisms that cause COVID-19-induced autoimmunity. The first theory proposes that molecular mimicry between the virus and some human proteins causes cytokine storms and the formation of autoantibodies. The role of antigen cross-reactivity is significant in SARS-CoV-2 infections, where it results in autoinflammatory dysregulation and subsequent tissue damage driven by the viral spike protein [19]. Furthermore, a heptapeptide sequence shared between the human proteome and the viral spike glycoprotein has been found [20]. Consequently, it is reasonable to assume that antibodies targeting the SARS-CoV-2 spike protein may recognize cross-reacting self-antigens or shared antigenic protein sequences. Another theory is on epitope spreading. Some epitopes remain unexposed or are expressed in low quantities. During the maturation of central immune organs, lymphocytes specific to autoantigens can bypass negative selection and join the mature lymphocyte pool [21]. Following SARS-CoV-2 infection, epitope spreading occurs, broadening the range of epitopes recognized by B- and T-cells. Endocytic processing, antigen presentation, and somatic hypermutation contribute to the sustained and worsening autoimmune damage in the liver [22], which can result in the onset of AIH. The other theory is called bystander activation and autoreactive lymphocytes. Following SARS-CoV-2 infection, cytokines such as tumor necrosis factor *α*, interferon *γ*, and interleukins 1β, 6, and 10 may be released, activating autoreactive B- and T-cells and causing autoimmune liver injury [23]. In a patient with post-SARS-CoV-2 vaccine AIH, spike-specific CD8 T-cells induced by SARS-CoV-2 were found in the peripheral blood along with extensive CD8 liver infiltration [17]. This observation supports the hypothesis that autoreactive lymphocytes are involved in the pathogenesis of AIH.

Many AIH patients respond successfully to the standard treatment with prednisolone alone or in combination with azathioprine, while some cannot tolerate it or relapse [1, 2]. In our case, following the administration of methylprednisolone in Sanandaj, there was a relative improvement in the total bilirubin level, but it rose again after a few days. Meanwhile, progressive bicytopenia was occurring. On one hand, corticosteroids could not be stopped due to worsening of liver enzymes and the possibility of the progression to acute liver failure, but on the other hand, they were causing severe immunosuppression and due to the progression of leukopenia, the patient was becoming more susceptible to opportunistic infections. Therefore, the pediatric gastroenterologist of Besat Hospital decided to refer the patient to Shiraz, Iran, for better evaluation and probable liver transplant.

There are two potential causes of bicytopenia in this case: COVID-19 and azathioprine. The most common COVID-19-induced hematological abnormalities in adults include lymphopenia, leukopenia, thrombocytopenia, disseminated intravascular coagulation, and a prothrombotic state. The proposed theory behind lymphopenia is that SARS-CoV-2 may directly infect lymphocytes via the expressed angiotensin-converting enzyme 2 (ACE2) receptors on their surface. In children, however, most of the infected patients have normal WBC counts, with leukopenia being the most common abnormality. Lower development of ACE2 may be the cause of uncommon lymphopenia among children. Most published studies show that the RBC system is unaffected in patients with coronavirus disease 2019. Another probable cause of cytopenia is autoimmunity. In immune-mediated leukopenia, autoantibodies against cell membrane antigens cause leukocyte destruction in the periphery. Another possibility is autoimmune hemolytic anemia, in which erythrocytes are destructed by autoreactive antibodies [24].

Azathioprine, a purine antimetabolite and immunosuppressive drug, is known to cause myelosuppression and pancytopenia, particularly in those with reduced levels of thiopurine methyltransferase (TPMT) activity [25]. The time from the initiation of the starting dose to the onset of marrow suppression varied from 10 days to 11 years in the literature [26, 27]. In our case, bicytopenia developed 16 days after azathioprine administration, just 2 days before the transfer to Namazi Hospital in Shiraz. This, along with the patient's lack of response to corticosteroids, ultimately convinced the physician in Sanandaj to proceed with the transfer. While it is difficult to determine whether COVID-19 or azathioprine caused the bicytopenia, the resolution of bicytopenia following IVIg administration suggests that IVIg may help mitigate the adverse effects of COVID-19, such as bicytopenia, in this patient.

In this case, we do not intend to suggest that COVID-19 is the cause of AIH. Rather, our focus is on the point that when COVID-19 is concurrent with AIH, IVIG may be beneficial, especially in cases with unresponsiveness to corticosteroids. While corticosteroids may have influenced the patient's improvement, their effects are generally expected to occur more rapidly. Considering the timing of the observed response following IVIg initiation, we believe IVIg played the primary role, with azathioprine likely requiring a longer duration to show its full impact. Additionally, the use of IVIg in corticosteroid-resistant AIH warrants further investigation and could be evaluated in future randomized controlled trials. To our knowledge, this is the first report on IVIg administration in an AIH patient with simultaneous COVID-19 infection, which could be owing to potential shortages and high costs of this medication.

The study has several limitations that should be acknowledged. Firstly, lack of long-term follow-up restricts the understanding of how IVIg therapy impacts patients over time, creating gaps in understanding its lasting efficacy and stability. Also, using retrospective data may have introduced bias, which could compromise the study's robustness. However, we comprehensively reviewed the available data to minimize its impact. Additionally, the possible classification of the AIH diagnosis, as determined by the scoring system, limits the generalizability and strength of the study's claims. Furthermore, the coexistence of various conditions obscures the ability to determine which is the main contributor to the symptoms and therapeutic responses, complicating the overall analysis.

Despite these limitations, we report this case to highlight the complexities and challenges of managing AIH in the context of concomitant COVID-19 infection. This unique presentation highlights the need for further research into tailored therapeutic strategies that address the interplay between these conditions.

## Figures and Tables

**Table 1:** Comparison of patient laboratory findings before and after IVIg administration.

	Before IVIg administration	2 days after	5 days after	9 days after	13 days after
AST	300	135	134	160	240
ALT	480	350	269	290	302
ALP	572	407	348	393	466
Total bilirubin	21	7.6	6	4.2	4
Direct bilirubin	15.7	5	2	2.5	2.1
Total protein	5.4	7.8	6.3	7.4	7.1
Albumin	3.5	2.9	2.7	3.7	3.7
Globulin	1.9	4.9	3.6	3.7	3.4

## Data Availability

The datasets supporting this case report are not publicly available to protect patient confidentiality. However, anonymized data can be made available upon reasonable request to the corresponding author.
